# Risk factors for placenta accreta spectrum disorders in women with any prior cesarean and a placenta previa or low lying: a prospective population-based study

**DOI:** 10.1038/s41598-024-56964-9

**Published:** 2024-03-19

**Authors:** Gilles Kayem, Aurélien Seco, Francoise Vendittelli, Catherine Crenn Hebert, Corinne Dupont, Bernard Branger, Cyril Huissoud, Jeanne Fresson, Norbert Winer, Bruno Langer, Patrick Rozenberg, Olivier Morel, Marie Pierre Bonnet, Franck Perrotin, Elie Azria, Lionel Carbillon, Coralie Chiesa, Pierre Raynal, René Charles Rudigoz, Sophie Patrier, Gaël Beucher, Michel Dreyfus, Loïc Sentilhes, Catherine Deneux-Tharaux

**Affiliations:** 1 Trousseau Hospital, APHP, Sorbonne University, Paris, France; 2 CRESS U1153, INSERM, Obstetrical, Perinatal and Pediatric Epidemiology (EPOPé) Research Team, Paris University, Paris, France; 3 Clinical Research Unit Necker Cochin, APHP, Paris, France; 4 Réseau de Santé en Périnatalité d’Auvergne, Clermont-Ferrand, France; 5 Université Clermont Auvergne, Clermont Auvergne INP, CHU Clermont-Ferrand, CNRS, Institut Pascal, 63000 Clermont-Ferrand, France; 6 APHP, Louis Mourier University Hospital, Colombes, France; 7 Réseau périnatal des Hauts de Seine, PERINAT92, 60 Rue du Général Leclerc, Issy-Les-Moulineaux, France; 8 University Claude Bernard Lyon 1, RESHAPE INSERM U1290, Univ. Lyon, 7425 Lyon, France; 9 Réseau Périnatal Aurore, Hospices Civils de Lyon, Hôpital de la Croix Rousse, Lyon, France; 10 Réseau « Sécurité Naissance - Naître Ensemble » des Pays-de-la-Loire, Nantes, France; 11 Maternité de la Croix Rousse, Lyon, France; 12 CHRU Nancy, Réseau Périnatal Lorrain, Nancy, France; 13 Service de Gynécologie Obstétrique HME Université de Nantes, NUN, INRA, UMR 1280, Phan, Université de Nantes, 44000 Nantes, France; 14 CHU de Strasbourg, Strasbourg, France; 15 CHI de Poissy, Poissy, France; 16 CHRU de Nancy, Nancy, France; 17 Anesthesia and Critical Care Department, Trousseau Hospital, APHP, Sorbonne University, Paris, France; 18 CHRU de Tours, Tours, France; 19 Maternity Unit, Paris Saint Joseph Hospital, DHU Risks in Pregnancy, Paris Descartes University, Paris, France; 20 Réseau Périnatal NEF Naître dans l’Est Francilien, Paris 13 University, Villetaneuse, France; 21 CH de Versailles, Site Andre Mignot, Versailles, France; 22 CHU de Rouen, Rouen, France; 23 Service de Gynécologie Obstétrique et Médecine de la Reproduction, CHU de Caen, Avenue Côte de Nacre, Caen Cedex 9, France; 24 Department of Obstetrics and Gynecology, Bordeaux University Hospital, Bordeaux, France

**Keywords:** Medical research, Epidemiology

## Abstract

This study aimed to identify the risk factors for placenta accreta spectrum (PAS) in women who had at least one previous cesarean delivery and a placenta previa or low-lying. The PACCRETA prospective population-based study took place in 12 regional perinatal networks from 2013 through 2015. All women with one or more prior cesareans and a placenta previa or low lying were included. Placenta accreta spectrum (PAS) was diagnosed at delivery according to standardized clinical and histological criteria. Of the 520,114 deliveries, 396 fulfilled inclusion criteria; 108 were classified with PAS at delivery. Combining the number of prior cesareans and the placental location yielded a rate ranging from 5% for one prior cesarean combined with a posterior low-lying placenta to 63% for three or more prior cesareans combined with placenta previa. The factors independently associated with PAS disorders were BMI ≥ 30, previous uterine surgery, previous postpartum hemorrhage, a higher number of prior cesareans, and a placenta previa. Finally, in this high-risk population, the rate of PAS disorders varies greatly, not only with the number of prior cesareans but also with the exact placental location and some of the women's individual characteristics. Risk stratification is thus possible in this population.

## Introduction

Placenta accreta spectrum (PAS) is characterized histologically by the total or partial absence of decidua and placental invasion into the myometrium^1,2^. PAS has become a leading cause of peripartum hysterectomy and is associated with major maternal morbidity and even maternal death worldwide^3,4^.

Maternal mortality and morbidity are lower in women with PAS disorders who deliver in a referral center with a multidisciplinary care team experienced in managing the surgical risks and perioperative challenges that these disorders present^5–9^. Transfer to a referral center, however, requires both recognition of the women at risk of PAS and accurate prenatal diagnosis. Although routine imaging examinations have improved PAS screening during pregnancy, their performance is not yet good enough to reliably detect or rule out all cases. Risk stratification based on clinical indicators would thus be useful in helping to identify subgroups of women at very high risk.

Overall, 30 to 65% of PAS cases occur in women with at least one prior cesarean and a placenta previa or low-lying^10–12^. The combination of these two factors has been identified as a profile at high risk for PAS, and prenatal screening for these disorders is usually performed among this subgroup of women, rather than among all pregnant women^12^.

A few studies have analyzed the risk factors for PAS specifically in women with this high-risk profile^13–16^. They have found that among women with prior cesareans and placenta previa the risk of PAS increases with the number of previous cesareans. These studies were limited, however, by their center-based design with women included who may not have been representative of the general population. They also did not explore other possible PAS risk factors, most importantly, precise placenta location, but also individual characteristics and details of obstetric history, although this information could provide additional tools to stratify the risk of PAS among these women.

This prospective population-based study was designed to include all women with any prior cesarean deliveries and a placenta previa or low lying^17^. Our objective was to apply a population-based approach to investigate the risk factors, particularly the precise placenta location, for PAS in women with this profile.

## Material and methods

This study took place in 176 French maternity units and hospitals belonging to 12 regional perinatal networks over a two-year period, from November 1, 2013, to October 31, 2015. It had a source population of 520 114 deliveries, 30% of the national total; the characteristics of the women giving birth were similar to those nationwide (Table S.1)^18^.

Recruitment and data collection started only after informed consent has been obtained from the participants of this study. The appropriate ethics committees approved this study.

The study protocol has previously been described^17^. In brief, all women with at least one previous cesarean section and a placenta previa or low-lying (0–20 mm from the internal cervical os, considered posterior if predominantly posterior or anterior if predominantly anterior), diagnosed by the last ultrasound before delivery-were included in the immediate postpartum period (i.e., a live or stillborn baby after 22 weeks of gestation). Placentas previa were considered as a single category, regardless of the predominant position of the placenta. First-, second-, and third-trimester ultrasound examinations are free of charge and part of routine care in France. Therefore nearly all pregnant women have all three scans; when a placenta is found to be previa or low lying, a transvaginal ultrasound is generally performed before delivery, in accordance with French recommendations^18^.

All women meeting the inclusion criteria were identified and flagged by local coordinators at each participating center. Moreover, the exhaustiveness of the case identification was verified by clinical research midwives, who consulted the delivery logbook and the computerized databases at each center. All women were followed up until their post-delivery discharge, and those with PAS until 6 months postpartum.

PAS was defined by the presence of at least one of the following clinical or histological criteria:^17^ (1) manual removal of the placenta partially or totally impossible and no cleavage plane between part or all of the placenta and the uterus; (2) massive bleeding from the implantation site after forced placental removal in the absence of another cause of postpartum hemorrhage (PPH); (3) histological confirmation of PAS on a hysterectomy specimen; (4) sign of PAS at laparotomy corresponding to the Grade 2 (increta type of abnormally invasive placenta) and 3 (percreta type of abnormally invasive placenta) clinical criteria of the FIGO classification^19^.

Research midwives manually reviewed the medical files to collect the following information: (1) maternal characteristics and medical history, including the women's age, geographical origin, medical insurance coverage, smoking status during pregnancy, body mass index, pre-existing diabetes or hypertension, gravidity, parity and any history of the following: PPH, manual removal of a placenta, termination of pregnancy, uterine surgery (myomectomy, hysteroscopy, or curettage), and cesarean delivery; (2) characteristics of the current pregnancy, including use of in vitro fertilization (IVF), type of pregnancy (singleton or multiple), pregnancy complications (second- or third-trimester bleeding, gestational hypertension, preeclampsia or preterm premature rupture of the membranes), and placental location.

### Statistical analysis

The population-based incidence of women with a placenta previa or low lying and at least one prior cesarean was calculated with its 95% confidence interval (95% CI). Characteristics of women, pregnancies, deliveries and pregnancy outcomes were described and compared between women with and without PAS. Categorical variables were compared with Pearson's Chi2 test or Fisher's exact test, and quantitative variables with Student’s t-test or Wilcoxon's rank-sum test, as appropriate.

Risk factors for PAS were identified by univariate and multivariable logistic regression modelling. Variables included in the multivariable model were selected based on the available literature and the results of the univariate analysis. We used multilevel modelling to take the correlation between observations within perinatal networks into account. The proportion of women with missing data for any covariate included in the multivariable model ranged from 0–8%; 333 (84%) women had no missing data. Missing data were considered missing at random. We imputed them by chained equations and computed 30 imputation datasets.

We used Stata/SE 15.0 (StataCorp LP, College Station, TX) to analyze the data.

### Ethical approval

The appropriate ethics committees (Consultative Committee on the Treatment of Data on Personal Health for Research Purposes—reference n° AOR12156; 12/ 19/2013 , and the Committee for the Protection of People Participating in Biomedical Research—reference CPP 13-017; 05/28/2013) , and the National Data Protection Authority (CNIL n° DR-2013-427; 12/20/2013) approved the study.

## Results

Of the 520 114 women who gave birth, 396 (0.76‰; 95% CI 0.69–0.84) had at least one prior cesarean delivery and a placenta previa or low-lying. Among them, 108 (27%; 95% CI 23–32) had PAS. Table S.2 shows the distribution of the PAS criteria: 83% of PAS cases met more than one of these criteria.

Women with PAS more often had a BMI≧30, higher gravidity and parity, a previous PPH, a previous PAS disorder, more prior cesareans, previous uterine surgery excluding cesareans, and more frequent previa locations of the placenta (Table 1).

**Table 1:** Characteristics of women with any prior cesareans and an abnormally located placenta, overall and according to the presence or absence of placenta accreta spectrum (PAS).

Variable	Total		No PAS		PAS		*P*
	N = 396		N = 288		N = 108		
	n	%	n	%	n	%	
Maternal characteristics							
Age (years)							.15
< 30	53	(14)	41	(14)	12	(11)	
30–34	134	(34)	88	(31)	46	(43)	
35–39	144	(36)	108	(37)	36	(33)	
≧ 40	65	(16)	51	(18)	14	(13)	
Geographical origin							.46
France/Europe	227	(62)	170	(64)	57	(56)	
North Africa	63	(17)	41	(16)	22	(22)	
Sub-Saharan Africa	56	(15)	40	(15)	16	(16)	
Asia/other	19	(5)	13	(5)	6	(6)	
Missing data	31	(8)	24	(8)	7	(6)	
BMI (kg/m^2^)							.02
< 25	179	(48)	134	(49)	45	(43)	
25–29.9	118	(31)	90	(33)	28	(27)	
≧30	79	(21)	47	(17)	32	(30)	
Missing data	20	(5)	17	(6)	3	(3)	
Gravidity*							.001
2	80	(20)	70	(24)	10	(9)	
≧3	316	(80)	218	(76)	98	(91)	
Parity*							< .001
2	173	(44)	144	(50)	29	(27)	
3	124	(31)	86	(30)	38	(35)	
≧4	99	(25)	58	(20)	41	(38)	
Past history							
Pre-existing hypertension	11	(3)	9	(3)	2	(2)	.73
Pre-existing diabetes	8	(2)	4	(1)	4	(4)	.22
Previous postpartum hemorrhage	39	(10)	20	(7)	19	(18)	.002
Previous manual removal of the placenta	8	(2)	5	(2)	3	(3)	.45
Previous placenta previa	37	(9)	28	(10)	9	(8)	.67
Previous cesarean							< .001
1	266	(67)	216	(75)	50	(46)	
2	75	(19)	48	(17)	27	(25)	
≧3	54	(14)	23	(8)	31	(29)	
Any previous uterine surgery other than cesarean (including endouterine aspiration)	114	(29)	73	(25)	41	(38)	.01
Current pregnancy							
IVF	15	(4)	13	(5)	2	(2)	.37
Multiple pregnancy	12	(3)	11	(4)	1	(1)	.19
Smoking during pregnancy	73	(19)	48	(17)	25	(25)	.11
*Missing data*	15	(4)	9	(3)	6	(6)	
Bleeding during 2^nd^ and 3^rd^ trimester	244	(63)	174	(61)	70	(67)	.33
Missing data	7	(2)	4	(1)	3	(3)	
PPROM	35	(9)	28	(10)	7	(6)	.31
Gestational hypertension/pre-eclampsia	12	(3)	8	(3)	4	(4)	.64
Placenta location							< .001
Previa	264	(67)	176	(61)	88	(81)	
Anterior low-lying	66	(17)	52	(18)	14	(13)	
Posterior low-lying	66	(17)	60	(21)	6	(6)	

PAS: placenta accreta spectrum; BMI: Body mass index; IVF: in vitro fertilization; PPROM: preterm premature rupture of membranes. Except when specified, the rate of missing data was less than 1%. Percentages of missing data were calculated among all women; other percentages were calculated among women with no missing data.

*Gravidity and parity were at least two because by definition, all women in the study population had at least one previous cesarean birth and the present birth.

The PAS rates in women with one, two, and three or more prior cesareans were respectively, 19% (95% CI 14–24), 36% (95% CI 25–48), and 57% (95% CI 43–71). PAS rates by placental position were 9% (95% CI 3–19) when it was posterior low lying, 21% (95% CI 12–33) when anterior low lying, and 33% (95% CI 28–39) when previa (Table 2). Combining the number of prior cesareans and the location of the placenta yielded a PAS rate ranging from 5% (95% CI 1–15) for one prior cesarean combined with a posterior low-lying placenta, to 63% (95% CI 45–79) for three or more prior cesareans combined with placenta previa (Table 2).

**Table 2:** Rate of placenta accreta spectrum according to the number of previous cesareans and the placenta location (N = 396).

Number of previous cesareans	Placenta location			
	Posterior low lying	Anterior low lying	Previa	Total
	n with PAS/N % (95%CI)	n with PAS/N % (95%CI)	n with PAS/N % (95%CI)	n with PAS/N % (95%CI)
**1**	2/44	4/42	44/180	50/266
	5 (1–15)	10 (3–23)	24 (18–31)	19 (14–24)
**2**	2/17	3/10	22/48	27/75
	12 (1–36)	30 (7–65)	46 (31–61)	36 (25–48)
3 or more	2/5	7/14	22/35	31/54
	40 (5–85)	50 (23–77)	63 (45–79)	57 (43–71)
*Not known*	*–*	*–*	*0/1*	*0/1*
Total	6/66	14/66	88/264	108/396
	9 (3–19)	21 (12–33)	33 (28–39)	27 (23–32)

PAS: placenta accreta spectrum.

In the multivariable analysis, the risk factors for PAS among these women with any prior cesarean delivery and an abnormally located placenta were BMI ≧ 30, previous uterine surgery, previous PPH, more prior cesareans and placenta previa (Table 3). The rate of PAS was 2% (95% CI 0–11) in women without any of these four risk factors and 31% (95% CI 26–36) in women with at least one.

**Table 3:** Risk factors for placenta accreta spectrum in women with any prior cesarean deliveries and an abnormally located placenta.

	cOR (95% CI)	aOR* (95% CI)
Age		
< 29	1	1
30–34	1.82 (0.87–3.81)	1.73 (0.74–4.03)
35–39	1.14 (0.54–2.43)	0.83 (0.35–1.98)
≥ 40	0.98 (0.40–2.38)	0.67 (0.23–1.93)
Geographical origin		
France/Europe	1	1
North Africa	1.63 (0.88–3.03)	1.45 (0.69–3.04)
Sub-Saharan Africa	1.18 (0.61–2.30)	0.94 (0.43–2.07)
Others	1.43 (0.51–3.97)	1.82 (0.56–5.94)
BMI		
< 30	1	1
≥ 30	2.03 (1.21–3.41)	1.94 (1.05–3.59)
Smoking during pregnancy		
No	1	1
Yes	1.55 (0.90–2.69)	1.66 (0.86–3.22)
Previous uterine surgery†		
No	1	1
Yes	1.80 (1.12–2.89)	1.82 (1.03–3.21)
Parity		
2	1	1
3	2.20 (1.26–3.84)	1.39 (0.67–2.88)
≥ 4	3.66 (2.05–6.52)	1.07 (0.44–2.63)
Prior cesarean		
1	1	1
2	2.40 (1.36–4.23)	2.34 (1.10–5.00)
≥ 3	6.04 (3.21–11.37)	6.41 (2.48–16.59)
Previous PPH		
No	1	1
Yes	2.85 (1.44–5.62)	2.90 (1.31–6.42)
Location of placenta		
Posterior Low Lying	1	1
Anterior Low-lying	2.83 (1.00–7.99)	2.29 (0.73–7.15)
Previa	5.12 (2.12–12.38)	5.15 (1.99–13.30)

PAS: placenta accreta spectrum; BMI: body mass index; PPH: postpartum hemorrhage; cOR crude odds ratio; aOR adjusted odds ratio;

*Multilevel multivariate logistic regression model adjusted for age, geographical origin, BMI, smoking during pregnancy, previous uterine surgery, parity, previous cesarean, previous PPH, and location of placenta. Multiple imputation by chained equations was used for missing data; † Previous endouterine aspiration, uterine synechiae, myomectomy, surgical hysteroscopy or other uterine surgery.

## Discussion

About one quarter of the women with any prior cesareans and a placenta previa or low lying had PAS. Risk factors for these disorders in this population were BMI ≧ 30, previous uterine surgery, previous PPH, prior cesareans (risk increasing with number), and placenta previa versus low-lying.

The 27% PAS rate among this high-risk profile population contrasts with the proportion of 5 to 10% reported in two previous population-based studies^10,11^. Potential explanations for this difference may be related to the fact that those two studies did not collect the total number of women with abnormally located placentas specifically for the study, as we did, but instead derived it from routine databases where diagnoses may be less accurately reported. They may also have included in their denominator previa or low-lying placentas in the second but not the third trimester and women with prenatal bleeding from other causes^10,11^.

Several previous studies have explored PAS risk factors. Most were conducted in general populations of pregnant women and compared women with and without PAS. One or more prior cesareans and an abnormally located placenta are both present in approximately half the cases of PAS, and their combination has been recognized as a major risk factor^10,11,13–16^. These findings have guided current clinical practice to focus PAS screening in the subgroup of women combining these conditions. However, the characteristics of women with PAS have been shown to differ between those with the combination of at least one prior cesarean and an abnormally located placenta and those without both^12,20^. Risk factors for PAS may therefore be specific to each of these two subgroups and should be explored separately. No previous study has specifically assessed the risk factors for PAS among women with any prior cesareans and placenta previa or low-lying.

We found that the risk of PAS increases not only with the number of prior cesareans but also varies with the placenta location relative to the internal cervical os: despite findings about the risk of PAS and the number of previous cesareans, no study had explored the risk according to the placenta location^14^. Posterior low-lying placenta are generally not implanted on the cesarean scar which may explain the lower risk of PAS. The position of the previous uterine cesarean scar (or scars) may explain the higher risk of PAS in women with placenta previa compared with low-lying locations. Cesarean scars are located in the anterior uterine wall, close to the internal os, or in the cervix; their precise position depends on cervical dilation at the time of the previous cesareans^21^. In an ongoing pregnancy with a previous cesarean, the scar is located in the cervix in 50 to 75% of cases^22^.

We found higher BMI to be associated with a higher risk of PAS in women with prior cesareans and placenta previa or low-lying. The growing proportion of pregnant women who are obese may thus contribute to the increasing rate of PAS disorders, along with rising cesarean rates^23,24^. This association is found inconsistently in the literature in the general population and had never been studied within the high risk population of women with any prior cesareans and placenta previa or low-lying^10,11^. Obesity may contribute to abnormal decasualization through various mechanisms involving hormonal balance and the effects of endometrial free fatty acid accumulation and lipotoxicity^25^.

PAS was also more frequent in women with previous PPH, which may be a cause or a marker of decidualization failure and has been associated with PAS in general populations of parturients^11,26^. Our study shows, interestingly, that even among women with a prior cesarean and placenta previa or low-lying, previous PPH is associated with an increased risk of PAS.

Strengths of our study include its large size, its population-based design with a rigorous verification of case ascertainment completeness, and especially its focus on women with the two best-known determinants of PAS, which provides a clinically relevant population. Another strength, in contrast with previous studies^13–16^, is the precise analysis of the placenta location and the availability of detailed data regarding the distance between the placental edge and the internal cervical os.

Contrary to other studies, we did not find any association of IVF with PAS in our study^27–30^. However, previous studies who reported an increased risk of PAS in women with IVF were mostly conducted in a general population of pregnant women**,** whereas our study was focused on the specific population of women with a placenta previa and a previous cesarean^31^. An important point is that IVF is associated with a higher risk of placenta previa which itself is a major risk factor of PAS^30^. Therefore, the increased risk of PAS in cases of IVF is likely, at least in part, mediated by the increased risk of placenta previa**,** but that, among women with previa and previous cesarean, having or not having had an IVF does not make a difference in terms of PAS risk. Further studies are needed to confirm this hypothesis.

The specific identification of risk factors for PAS in women with any prior cesareans and an abnormally located placenta may be useful for pinpointing women at particularly high risk of PAS to customize the information they receive as well as their care during pregnancy and delivery.

This study suggests a pathophysiological hypothesis related to abnormal decidualization that should be investigated to find specific targets for preventing PAS disorders. In addition, in women with any prior cesareans and an abnormally located placenta, a next step would be to test the predictive performance of combining the clinical risk factors identified here and imaging features in a single score.

One potential limitation is the absence of histologic confirmation of PAS for the cases without hysterectomies. The prospective design and prespecified definitions make the risk of false diagnosis of PAS low. Another potential limitation is that the number of women with a posterior low-lying placenta may have been underestimated if some that were asymptomatic were not identified. That could overestimate the prevalence of PAS among this subgroup but is unlikely to bias the risk factors of PAS identified.

Finally, the rate of PAS disorders varies greatly not only with the number of prior cesareans but also with the precise location of the placenta and some of the women's individual characteristics. The specific identification of risk factors for PAS in women with any prior cesareans and a placenta previa or low lying will help to pinpoint women at particularly high risk of PAS to customize the information they receive as well as their care during pregnancy and delivery.

### Supplementary Information

Supplementary Tables.

## Data Availability

Data are available if requested to Gilles Kayem after acceptance by our institution.

## References

[1] Khong TY, Robertson WB (1987). Placenta creta and placenta praevia creta. Placenta.

[2] Jauniaux E, Jurkovic D (2012). Placenta accreta: Pathogenesis of a 20th century iatrogenic uterine disease. Placenta.

[3] van den Akker T, Brobbel C, Dekkers OM, Bloemenkamp KW (2016). Prevalence, indications, risk indicators, and outcomes of emergency peripartum hysterectomy worldwide: A systematic review and meta-analysis. Obstet. Gynecol..

[4] Saucedo M, Deneux-Tharaux C, Bouvier-Colle MH (2013). Ten years of confidential inquiries into maternal deaths in France, 1998–2007. Obstet. Gynecol..

[5] Eller AG, Bennett MA, Sharshiner M (2011). Maternal morbidity in cases of placenta accreta managed by a multidisciplinary care team compared with standard obstetric care. Obstet. Gynecol..

[6] Shamshirsaz AA, Fox KA, Erfani H (2017). Multidisciplinary team learning in the management of the morbidly adherent placenta: Outcome improvements over time. Am. J. Obstet. Gynecol..

[7] Al-Khan A, Gupta V, Illsley NP (2014). Maternal and fetal outcomes in placenta accreta after institution of team-managed care. Reprod. Sci..

[8] Silver RM, Fox KA, Barton JR (2015). Center of excellence for placenta accreta. Am. J. Obstet. Gynecol..

[9] Erfani H, Fox KA, Clark SL (2019). Maternal outcomes in unexpected placenta accreta spectrum disorders: single-center experience with a multidisciplinary team. Am. J. Obstet. Gynecol..

[10] Fitzpatrick KE, Sellers S, Spark P, Kurinczuk JJ, Brocklehurst P, Knight M (2012). Incidence and risk factors for placenta accreta/increta/percreta in the UK: A national case-control study. PLoS ONE.

[11] Thurn L, Lindqvist PG, Jakobsson M (2016). Abnormally invasive placenta-prevalence, risk factors and antenatal suspicion: Results from a large population-based pregnancy cohort study in the Nordic countries. BJOG.

[12] Kayem G, Seco A, Beucher G (2021). Clinical profiles of placenta accreta spectrum: The PACCRETA population-based study. BJOG.

[13] Miller DA, Chollet JA, Goodwin TM (1997). Clinical risk factors for placenta previa-placenta accreta. Am. J. Obstet. Gynecol..

[14] Silver RM, Landon MB, Rouse DJ (2006). Maternal morbidity associated with multiple repeat cesarean deliveries. Obstet. Gynecol..

[15] Bowman ZS, Eller AG, Bardsley TR, Greene T, Varner MW, Silver RM (2014). Risk factors for placenta accreta: A large prospective cohort. Am. J. Perinatol..

[16] Clark SL, Koonings PP, Phelan JP (1985). Placenta previa/accreta and prior cesarean section. Obstet. Gynecol..

[17] Kayem G, Deneux-Tharaux C, Sentilhes L, Group P (2013). PACCRETA: Clinical situations at high risk of placenta ACCRETA/percreta: Impact of diagnostic methods and management on maternal morbidity. Acta Obstet. Gynecol. Scand..

[18] Enquête nationale périnatale (2016), Paris, France, http://www.epope-inserm.fr/wp-content/uploads/2017/10/ENP2016_rapport_complet.pdf. Paris, France, http://www.epope-inserm.fr/wp-content/uploads/2017/10/ENP2016_rapport_complet.pdf., 2016 (vol 2019).

[19] Jauniaux E, Ayres-de-Campos D, Langhoff-Roos J, Fox KA, Collins S (2019). FIGO classification for the clinical diagnosis of placenta accreta spectrum disorders. Int. J. Gynaecol. Obstet..

[20] Hessami K, Salmanian B, Einerson BD (2022). Clinical correlates of placenta accreta spectrum disorder depending on the presence or absence of placenta previa: A systematic review and meta-analysis. Obstet. Gynecol..

[21] Kamel R, Eissa T, Sharaf M, Negm S, Thilaganathan B (2021). Position and integrity of uterine scar are determined by degree of cervical dilatation at time of Cesarean section. Ultrasound Obstet. Gynecol..

[22] Markovitch O, Tepper R, Hershkovitz R (2013). Sonographic assessment of post-cesarean section uterine scar in pregnant women. J. Matern. Fetal. Neonatal. Med..

[23] Fisher SC, Kim SY, Sharma AJ, Rochat R, Morrow B (2013). Is obesity still increasing among pregnant women? Prepregnancy obesity trends in 20 states, 2003–2009. Prev. Med..

[24] Heslehurst N, Ells LJ, Simpson H, Batterham A, Wilkinson J, Summerbell CD (2007). Trends in maternal obesity incidence rates, demographic predictors, and health inequalities in 36,821 women over a 15-year period. Bjog.

[25] Rhee JS, Saben JL, Mayer AL (2016). Diet-induced obesity impairs endometrial stromal cell decidualization: A potential role for impaired autophagy. Hum. Reprod..

[26] Eshkoli T, Weintraub AY, Sergienko R, Sheiner E (2013). Placenta accreta: Risk factors, perinatal outcomes, and consequences for subsequent births. Am. J. Obstet. Gynecol..

[27] Nagata C, Yang L, Yamamoto-Hanada K (2019). Complications and adverse outcomes in pregnancy and childbirth among women who conceived by assisted reproductive technologies: A nationwide birth cohort study of Japan environment and children's study. BMC Pregnancy Childbirth.

[28] Tanaka H, Tanaka K, Osato K (2020). Evaluation of maternal and neonatal outcomes of assisted reproduction technology: A retrospective cohort study. Medicina (Kaunas).

[29] Zhu L, Zhang Y, Liu Y (2016). Maternal and live-birth outcomes of pregnancies following assisted reproductive technology: A retrospective cohort study. Sci. Rep..

[30] Carusi DA, Gopal D, Cabral HJ, Racowsky C, Stern JE (2023). A risk factor profile for placenta accreta spectrum in pregnancies conceived with assisted reproductive technology. F S Rep..

[31] Matsuzaki S, Nagase Y, Takiuchi T (2021). Antenatal diagnosis of placenta accreta spectrum after in vitro fertilization-embryo transfer: A systematic review and meta-analysis. Sci. Rep..

