# Supplementary material for: Risk factors for placenta accreta spectrum disorders in women with any prior cesarean and a placenta previa or low lying: a prospective population-based study
Source: Sci Rep. 2024 Mar 19;14:6564. doi: 10.1038/s41598-024-56964-9 (PMC10951207; doi:10.1038/s41598-024-56964-9)
Supplement: Supplementary file 1 — Supplementary Tables. [file 41598_2024_56964_MOESM1_ESM.docx]

**Supplemental material**

Table S1: Characteristics of parturient in France, comparison of women from PACCRETA regions versus other regions (National Perinatal Survey*).

|  | PACCRETA regions  N=4230  n (%) | | Other regions in France  N=8315  n (%) | |
| --- | --- | --- | --- | --- |
| Age  <35  35-39  >=40 | 3304  748  178 | (78.1)  (17.7)  (4.2) | 6590  1408  317 | (79.3)  (16.9)  (3.8) |
| Country of birth  France and Europe  North Africa  Sub-Saharan Africa  Others | 3228  299  241  128 | (82.9)  (7.7)  (6.2)  (3.3) | 6806  527  309  224 | (86.6)  (6.7)  (3.9)  (2.9) |
| BMI  <25  25-29.9  ≥30 | 2654  754  424 | (69.3)  (19.7)  (11.1) | 5255  1558  944 | (67.7)  (20.1)  (12.2) |
| Parity  0  1  2  ≥3 | 1778  1537  580  333 | (42.1)  (36.3)  (13.7)  (7.9) | 3540  2938  1204  636 | (42.6)  (35.3)  (14.5)  (7.6) |
| Smoking before pregnancy | 1078 | (27.7) | 2446 | (31.2) |
| Previous cesarean among multiparous women | 508/2448 | (20.7) | 923/4775 | (19.3) |
| IVF | 280 | (7.2) | 525 | (6.8) |
| Multiple pregnancy | 80 | (1.8) | 150 | (1.7) |
| Hypertension or preeclampsia during pregnancy | 187 | (4.4) | 353 | (4.3) |
| Abnormally located placenta | 43 | (1.0) | 90 | (1.1) |
| Cesarean delivery | 862 | (20.4) | 1596 | (19.2) |
| Preterm birth | 299 | (7.0) | 621 | (7.3) |
| Maternal transfer to ICU | 19 | (0.5) | 36 | (0.4) |

* 2016 National Perinatal Survey, available at <http://www.epopé-inserm.fr/wp> content/uploads/2017/11/ENP2016_rapport_complet.pdf

Table S2: Criteria for diagnosis of placenta accreta spectrum.

|  | PAS  N=108 | |
| --- | --- | --- |
|  | **n** | **%** |
| At least one criterion for PAS | 108 | (100) |
| Specific criteria |  |  |
| Manual removal of the placenta partially or totally impossible and no cleavage plane between part or all the placenta and the uterus | 68 | (63) |
| Massive bleeding from the implantation site after forced placental removal in the absence of another cause of postpartum hemorrhage | 50 | (46) |
| Histological confirmation of PAS on a hysterectomy specimen^*^ | 60 | (100) |
| Signs of PAS at laparotomy | 84 | (78) |
| Number of criteria for PAS |  |  |
| 1 | 18 | (17) |
| ≥2 | 90 | (83) |

^*^ Among women who had a hysterectomy
